# Supplementary material for: The implicated clinical factors for outcomes in 304 patients with salivary duct carcinoma: Multi‐institutional retrospective analysis in Japan
Source: Head Neck. 2022 Mar 29;44(6):1430–41. doi: 10.1002/hed.27034 (PMC9311811; doi:10.1002/hed.27034)
Supplement: Supplementary file 1 — Figure S1 (A) Typical histology of a de novo (CXPA[−]) case showing Roman bridge structures of large atypical cells with an eosinophilic cytoplasm, and comedonecrosis (hematoxylin and eosin stain). (B) Typical histology of a CXPA(+) case showing the co‐existence of a pleomorphic adenoma (PA) circumscribed with a fibrous capsule (yellow dotted line). The intracapsular component (IC) showed the growth of atypical glandular cells within the PA component, whereas the invasive component (Inv) showed the extracapsular growth of SDC cells (hematoxylin and eosin stain). Figure S2 Cumulative incidence of relapse (CIR) (A), cumulative incidence of local relapse (CILR) (B), cumulative incidence of cervical lymph node relapse (CICLNR) (C), and cumulative incidence of distant metastasis relapse (CIDMR) (D). The non‐dotted line represents each incidence and dotted lines show the 95% confidence interval. Table S1 The summary of postoperative therapy (POT). Table S2 Univariate and multivariate analyses for cumulative incidence of local relapse (CILR) and cumulative incidence of cervical lymph node metastasis (CICLNM). [file HED-44-1430-s001.pdf]

Head and Neck

The implicated clinical factors for outcomes in 304 patients with salivary duct carcinoma: A multi-institutional retrospective analysis in Japan

Journal:	*Head & Neck*
Manuscript ID	HED-21-0246.R3
Wiley - Manuscript type:	Original Article
Date Submitted by the Author:	18-Dec-2021
Complete List of Authors:	Kusafuka, Kimihide; Shizuoka General Hospital, Department of Pathology<br>Sato, Yoko; Shizuoka General Hospital, Division of Clinical Biostatics, Research Support Center<br>Nakatani, Eiji; Shizuoka General Hospital, Division of Clinical Biostatics, Research Support Center<br>Baba, Satoshi; Hamamatsu University School of Medicine Hospital, Department of Diagnostic Pathology,<br>Maeda, Matsuyoshi; Toyohashi Municipal Hospital, Department of Clinical Pathology,<br>Yamahegi, Koji; Hyogo Medical College, Department of Pathology<br>Ueda, Kaori; Nagoya City University, Department of Pathology and Molecular Diagnostics<br>Inagaki, Hiroshi; Nagoya City University; Nagoya City University, Department of Pathology and Molecular Diagnostics<br>Otsuki, Yoshiro; Seirei Hamamatsu General Hospital, Department of Pathology,<br>Kuroda, Naoto; Kobe Kyoudou Hospital, Department of Diagnostic Pathology<br>Suzuki, Kensuke; Kansai Medical University Hirakata Hospital, Otolaryngology-Head and Neck surgery<br>Iwai, Hiroshi ; Kansai Medical University, Department of Otorhinolaryngology-Head and Neck Surgery<br>Imamura, Yoshiaki; University of Fukui Hospital, Division of Diagnostic Pathology/Surgical Pathology,<br>Itakura, Junya; Kurashiki Central Hospital, Department of Anatomic Pathology<br>Yamanaka, Shoji; Yokohama City University, Department of Diagnostic Pathology<br>Takahashi, Hideaki; Yokohama City University, School of Medicine, Otorhinolaryngology, Head and Neck Surgery<br>Ito, Ichiro; Nagano Red Cross Hospital, Department of Diagnostic Pathology<br>Akashi, Takumi; Tokyo Medical and Dental University, Division of Surgical Pathology<br>Daa, Tsutomu; Oita University School of Medicine Graduate School of Medicine, Department of Diagnostic Pathology,<br>Hamada, Mei; Saitama Medical University International Medical Center, Department of Diagnostic Pathology,

	Yasuda, Masanori; Saitama Medical University International Medical Center, Department of Pathology<br>Kawata, Ryo; Osaka Medical College, Department of Otorhinolaryngology-Head and Neck Surgery<br>Yamamoto, Hidetaka; Kyushu University, Anatomic Pathology<br>Tachibana, Yuri; Nagasaki University, Department of Pathology<br>Fukuoma, Junya; Nagasaki University<br>Muramatsu, Aya; Shizuoka General Hospital, Department of Pathology<br>Arai, Morikazu; Shizuoka General Hospital, Department of Pathology<br>Suzuki, Makoto; Shizuoka General Hospital, Department of Pathology

Key Words:	salivary duct carcinoma, outcomes, competing-risk model, Japanese patient, distant metastasis

SCHOLARONE™ Manuscripts

Clinical factors affecting outcomes of 304 patients with salivary duct carcinoma: A multi-institutional retrospective analysis in Japan

Kimihide Kusafuka, D.D.S., Ph.D.[1] (k-kusafuka@i.shizuoka-pho.jp); Yoko Sato, D.D.S., Ph.D.[2] (sato.yoko.shiz@gmail.com); Eiji Nakatani, Ph.D.[2] (nakatani.eiji.int@gmail.com); Satoshi Baba, M.D., Ph.D.[3] (baba@hama-med.ac.jp); Matsuyoshi Maeda, M.D., Ph.D.[4] (maeda-matsuyoshi@toyohashi-mh.jp); [5)]Koji Yamanegi, D.D.S, Ph.D.[5] (yamanegi@hyo-med.ac.jp); Kaori Ueda, D.M.D, Ph.D.[6] (kaori.u.0201@gmail.com); Hiroshi Inagaki, M.D., Ph.D.[6] (hinagaki@med.nagoya-cu.ac.jp); Yoshiro Otsuki, M.D., Ph.D.[7] (otsuki@sis.seirei.or.jp); Naoto Kuroda, M.D., Ph.D.[8] (kurochankochi@yahoo.co.jp); Kensuke Suzuki, M.D.[9] (suzukken@hirakata.kmu.ac.jp); Hiroshi Iwai, M.D., Ph.D.[9] (iwai@hirakata.kmu.ac.jp); Yoshiaki Imamura, M.D., Ph.D.[10] (suki@u-fukui.ac.jp); Junya Itakura, M.D., Ph.D.[11] (ji14830@kchnet.or.jp); Shoji Yamanaka, M.D., Ph.D.[12] (kowanyamanaka@gmail.com); Hideaki Takahashi, M.D.[13] (htk98@yokohama-cu.ac.jp); Ichiro Ito, M.D., Ph.D.[14] (ichirito@nifty.com); Takumi Akashi, M.D., Ph.D.[15] (akashi.path@tmd.ac.jp); Tsutomu Daa, M.D., Ph.D.[16] (daatom@med.oita-u.ac.jp); Mei Hamada, D.D.S., Ph.D.[17] (de421042@s.okayama-u.ac.jp), Masanori Yasuda, M.D.,

For Peer Review

2

Ph.D.[17] (m_yasuda@saitama-med.ac.jp); Ryo Kawata, M.D., Ph.D.[18] (oto034@osaka-med.ac.jp); Hidetaka Yamamoto, M.D., Ph.D.[19] (hidetaka@surgpath.med.kyushu-u.ac.jp); Yuri Tachibana, D.M.D., Ph.D.[20] (y.tachibana19890221@gmail.com); Junya Fukuoka, M.D., Ph.D.[20] (fukuokaj@nagasaki-u.ac.jp); Aya Muramatsu, M.D., Ph.D.[1] (aya-muramatsu@i.shizuoka-pho.jp); Kazumori Arai, M.D., Ph.D.[1] (m-arai@ny.tokai.or.jp); Makoto Suzuki, M.D., Ph.D.[1] (makoto-suzuki@i.shizuoka-pho.jp)

[1]Department of Pathology and [2]Division of Clinical Biostatistics, Research Support Center, Shizuoka General Hospital, Shizuoka, Japan, [3]Department of Diagnostic Pathology, Hamamatsu University School of Medicine Hospital, Shizuoka, Japan, [4]Department of Clinical Pathology, Toyohashi Municipal Hospital, Aichi, Japan, [5]Department of Pathology Hyogo Medical College, Hyogo, Japan, [6]Department of Pathology and Molecular Diagnostics, Nagoya City University, Nagoya, Japan, [7]Department of Pathology, Seirei Hamamatsu General Hospital, Shizuoka, Japan, [8]Department of Diagnostic Pathology, Kobe Koudou Hospital Center, Hyogo, Japan, [9]Department of Otorhinolaryngology-Head and Neck Surgery, Kansai Medical University, Osaka, Japan, [10]Division of Diagnostic Pathology/Surgical Pathology, University of Fukui Hospital, Fukui, Japan, [11]Department of Anatomic Pathology,

For Peer Review

3

Kurashiki Central Hospital, Okayama, Japan, [12]Department of Diagnostic Pathology, [13]Department of Otorhinolaryngology-Head and Neck Surgery, Yokohama City University Graduate School of Medicine, Kanagawa, Japan, [14]Department of Diagnostic Pathology, Nagano Red Cross Hospital, Nagano, Japan, [15]Division of Surgical Pathology, Tokyo Medical and Dental University Hospital, Tokyo, Japan; [16]Department of Diagnostic Pathology, Oita University, Oita, Japan, [17]Department of Diagnostic Pathology, Saitama Medical University International Medical Center, Saitama, Japan, [18]Department of Otorhinolaryngology-Head and Neck Surgery, Osaka Medical College, Osaka, Japan, [19]Department of Anatomic Pathology, Kyushu University, Fukuoka, Japan and [20]Department of Pathology, Nagasaki University, Nagasaki, Japan

Correspondence to: Dr. Kimihide Kusafuka, D.D.S., Ph.D.
Department of Pathology, Shizuoka General Hospital
4-27-1 Kita-ando, Aoi-ku, Shizuoka city, Shizuoka 420-8527, Japan
TEL.: +81-54-247-6111
E-mail: k-kusafuka@i.shizuoka-pho.jp

Brief running title: Clinical analysis of SDC in Japanese

For Peer Review

Keywords: salivary duct carcinoma, outcomes, competing-risk model, Japanese patient

Acknowledgments:

The authors thank Mr. Naofumi Ishikawa, Ms. Yoko Yamazaki, Mr. Tomohiro Iwasaki, Ms. Chinatsu Tsuchiya, Ms. Aki Kubota, Mr. Kensuke Shimazaki, Mr. Kazuki Hirata, Mr. Koji Takahashi, Ms. Nobuyo Tsujino, and Mr. Yosuke Saguchi, the co-medical staff of Department of Pathology, Shizuoka General Hospital, Shizuoka, Japan, for providing technical assistance. We are grateful to Ms. Mamiko Uemura, secretariat, Department of Pathology, Shizuoka General Hospital, Shizuoka, Japan.

For Peer Review

Author contributions

KK designed and drafted the manuscript, and KK, AM, KA, and MS made the histopathological diagnosis for the central pathological review. KK, SB, MM, KY, KU, HIn, YO, NK, KS, HIw, YI, JI, SY, HT, II, TA, TD, MH, YY, RK, and HY selected cases and provided samples with pathological and clinical data. KK, YS, and

EN performed statistical analyses of all data. MS supervised this manuscript. All of the authors have read and approved the final manuscript.

Funding

This work was supported in part by a Grant-in-Aid for Science Research from the Ministry of Education, Culture, Sports, Science and Technology of Japan (15K08351 to HIn) and a Grant-in-Aid for Medical Research Support Project of Shizuoka Prefectural Hospital Organization in 2019 of Japan (to KK).

Competing interests

The authors declare that they have no competing interests related to this study.

Ethical approval and consent to participate

The present study was approved by the Institutional Review Board of Shizuoka General Hospital (SGHIRB#2019007). All subjects signed informed consent forms before participating.

Availability of data and materials

The datasets used and analyzed during the present study are available from the corresponding author upon reasonable request.

For Peer Review

6

Abstract:

Background. Salivary duct carcinoma (SDC) is a high-grade salivary malignancy that frequently occurs as the carcinomatous component of carcinoma ex pleomorphic adenoma. We herein examined the clinical factors affecting outcomes in a large cohort of SDC.

Methods. We selected 304 SDC cases and investigated clinical characteristics and the factors affecting outcomes.

Results. The median age of the cases examined was 68 years, the most common primary site was the parotid gland (238 cases), and there was a male predominance (M/F=5:1). Outcomes were significantly worse when the primary tumor site was the minor salivary glands (SG) than when it was the major SG. Outcomes were also significantly worse in pN(+) cases (161 cases) than in pN0 cases, particularly those with a metastatic lymph node number ≥11. The cumulative incidence of relapse and distant metastases was significantly higher in stage IV cases than in stage 0-III cases.

Conclusions. The absolute number of lymph node metastases, higher stages, and the minor SG as the primary tumor site were identified as factors affecting the outcome of SDC.

For Peer Review

Introduction

Salivary duct carcinoma (SDC) is a high-grade malignant tumor of the salivary glands (SG) [1]. However, it frequently occurs as the carcinomatous component of carcinoma ex pleomorphic adenoma (CXPA) [2]. Although SDC shares histological similarities with invasive ductal carcinoma of the breast, it typically shows an apocrine phenotype, which differs from the immunophenotypes (estrogen receptor [ER]+ and/or progesterone receptor [PgR]+) of breast cancer; the majority of SDC cases were immunohistochemically negative for ER and/or PgR, but variably positive for the androgen receptor (AR) and gross cystic disease fluid protein-15 [1,3]. Boon et al. previously reported that the absolute number of positive lymph nodes (LN) was associated with a poor overall survival (OS) and distant metastasis-free survival (DMFS) in a multivariable analysis of patients presenting without distant metastases in the Netherlands [4]. In contrast, Otsuka et al. showed that an advanced N stage independently affected both OS and disease-free survival (DFS) [5]. Therefore, the present study investigated the clinical features of SDC and attempted to identify the clinical factors affecting outcomes in the largest cohort of SDC patients in Japan.

Materials and methods

For Peer Review

Case selection

We initially collected data on 392 cases of “SDC”, “CXPA”, and “adenocarcinoma” from the pathology files of 18 institutions and a set of consultation files (from K.K.) between 1992 and 2020. Among them, SDC cases, including CXPA cases, were extracted from the central diagnostic system by four expert pathologists (K.K., A.M., K.A., and M.S.: Supplemental Figure 1). The following clinical data were collected from the medical records of each institution: age, sex, site, treatments, TNM classification, pathological stage, outcome, and follow-up data. Tumors were staged according to the eighth edition of the TNM Classification of Malignant Tumours [6]. Hashimoto’s classification for T factors and pathological stages was used to stage CXPA [7]: intracapsular (IC), minimally invasive (MinI), and widely invasive (WI), based on the invasive distance from the fibrous capsule, with MinI being ≤2 mm from the fibrous capsule of a co-existing pleomorphic adenoma (PA) and WI >2 mm from the capsule

Statistical analysis

OS was measured from the date of diagnosis until death by any cause. Patients alive at the last known follow-up date were censored. The cumulative incidence of relapse

For Peer Review

(CIR) was defined as the number of cases in which local or regional recurrence or distant metastasis occurred after the primary surgery, regardless of which occurred first. Patients that were alive without disease at the last known follow-up examination were censored for the purposes of the DFS analysis. The cumulative incidence of distant metastasis relapse (CIDMR) was defined as the number of cases in which distant metastasis occurred after the primary surgery. Frequencies and percentages were used for categorical variables. Survival curves were estimated by the Kaplan-Meier method and cumulative incidence curves using a competing-risk model analysis with Grey's test when the competing-risk event was death [8,9]. A univariate Cox proportional hazards regression model or Fine-Grey proportional hazard regression model was used for comparisons of patient and tumor characteristics and survival. A multivariate Cox proportional hazards regression model or Fine-Grey proportion hazard regression model was then performed by adjusting variables with P-values <0.05 in the univariate analysis. Hazard ratios, 95% confidence intervals (CI), and corresponding P-values were calculated based on the Wald test. The variables used in regression models for the cumulative accumulation of the overall incidence, relapse incidence, late cervical LN metastasis (CLNM), and distant metastasis incidence included sex, age (categorical), the T-, N-, and M-status, pathological stage, number of positive LN (categorical), CXPA,

For Peer Review

and the primary tumor site. We also investigated the pattern of treatment failure, including locoregional recurrence and distant metastasis. Patients with metastatic disease at diagnosis and those with missing values for one or more of the variables were excluded from the multivariable analysis. Data were analyzed using R version 3.6.2 software (The R Foundation for Statistical Computing, Vienna, Austria).

Results

Patient and tumor characteristics

A central pathological review and preserved data led to the inclusion of 304 eligible SDC cases from 392 cases in the initial collection (Figure 1). Patient characteristics are shown in Table 1. Median age was 68 years (range: 27-91) and there was a male predominance (83%). Although the univariate analysis of OS showed poorer outcomes for males than for females, a significant difference was not observed in the multivariate analysis. The most common primary tumor site was the parotid gland in 238 out of 304 cases (78%), followed by the submandibular gland in 55 (18%), and then the sublingual gland (1 case), palate (5 cases), parapharynx (2 cases), buccal gland (1 case), nasal cavity (1 case), and intraoral minor SG (1 case). Sixty-nine cases (23%) had Tis and T1 as early cancer, whereas 71 (23%), 79 (26%), and 80 (26%) had T2, T3, and T4,

For Peer Review

11

respectively, as advanced cancer. CLNM was detected in 161 cases (53%) in the primary surgery. Distant metastases were detected in 19 cases (6.3%). Based on the histological origin, the 304 SDC cases selected for the present study comprised 122 (40%) of *de novo* SDC and 182 (60%) of SDC arising from PA (CXPA cases), including 47 of the IC subtype, 23 of the MinI subtype, and 112 of the WI subtype. Pathological stages were as follows: stages 0-I in 59 cases (20%), stages II and III in 78 (26%), and stage IV in 156 (51%).

The most frequent target organs for late distant metastases (n=93) were the lungs (61 cases: 66%), followed by bone (32 cases: 34%), the central nervous system (19 cases: 20%), including the brain, meninges, and spine, distant LN (13 cases: 14%), including the mediastinal, axillary, and/or abdominal LN, the liver (11 cases: 12%), skin (8 cases: 8.6%), and other organs (4 cases), including the thyroid gland, breast, tongue, and kidney.

Therapy

A total of 107 patients underwent surgery only, while 197 received post-operative radiotherapy [RT] (102 patients: 52%), adjuvant chemotherapy [Ch] (13 patients: 6.6%), adjuvant chemoradiotherapy [CRT] (70 patients: 36%), and additional surgery (5

For Peer Review

patients: 2.5%) after the primary surgery (Supplemental Table 1). After the primary surgery, 25, 30, and 93 patients showed local recurrence, late CLNM (regional relapse), and distant metastasis, respectively. Among 110 patients with recurrence, five underwent additional surgery, while 102, 11, and 70 received additional RT, Ch, and CRT, respectively. Only 3 out of 61 patients with lung metastases recovered from the status of being alive with disease to the status of being alive without disease with additional surgery and RT for metastatic lesion(s).

Clinical outcomes and survival analysis

The median follow-up period was 2.93 years (minimum-maximum: 0.01-21.70 years). At the time of the analysis, 149 patients were alive without disease, 66 died of disease, 38 were alive with disease, and 19 died of other causes. Kaplan-Meier curves for OS, DFS, and DMFS are shown in Figure 2. The cumulative incidence rates of 1- and 5-year relapse were 26.2% (95% confidence interval [CI], 20.7-32.1%) and 49.0% (95%CI 41.9-55.7%), respectively. The cumulative incidence rates of 1- and 5-year local relapse (CILR), CLNM (CICLNM), and CIDMR were 7.0% (95%CI, 4.2-10.8%) 12.0% (95% CI, 8-16.9%), and 7.0% (95% CI, 4.2-10.8), and 12.0% (95% CI, 8-

For Peer Review

16.9%), 20.3% (95% CI, 15.4-25.7%), and 41.6% (95% CI, 34.7-48.4%), respectively (Supplemental Figures 1 and 2).

Cumulative incidence curves stratifying prognostic factors identified by univariate and multivariate regression models are shown in Figures 3 and 4 and Supplemental Figures 2 and 3, whereas those analyzed by the Fine-Grey proportional hazards model are shown in Tables 2 and 3. OS was significantly worse in patients with a higher pathological stage and larger number of LN metastases ($p<0.001$: 0 vs 1-10 vs ≥11 cancer-positive nodes). On the other hand, no significant differences were observed in CIR, CILR, CICLNM, and CIDMR between *de novo* (CXPA[-]) and CXPA-WI cases, whereas OS, CIR, CILR, CICLNR, and CIDMR were better in CXPA-IC/MinI cases than in *de novo* and CXPA-WI cases. The multivariate analysis identified stage IV ($p<0.001$ vs. stages 0, I, II, and III, respectively) and ≥11 positive LN ($p=0.028$; vs. no LN metastasis) as independent prognostic factors for OS. In addition to stage IV, ≥11 positive LN ($p<0.001$; vs. no LN metastasis) and minor SG as the primary tumor site ($p<0.001$ and $p=0.003$; vs the parotid gland and submandibular gland, respectively) were identified as strongly independent factors for CIR. Similarly, minor SG as the primary tumor site ($p<0.001$ and $p=0.012$; vs. the parotid gland and submandibular gland, respectively), stage IVA/B ($p=0.005$; vs. stages 0, I, II, and III), and ≥11 positive

For Peer Review

LN (p<0.001; vs. no LN metastasis) were also independent prognostic factors for CIDMR.

Patterns of treatment failure

As shown in Figure 5A, treatment failure occurred in 110 cases (36%), including 25 (8.2%) local, 30 (9.9%) regional, and 93 (31%) distant failures, of which 65 (59%) were without locoregional failure. As shown in Figure 5B, the most common sites of distant metastasis were the lungs (n=61 cases), followed by bone (n=32 cases), the central nervous system (n=19 cases), distant LN (n=13 cases), the liver (n=11 cases), and skin (n=8 cases). Pre-operative distant metastases were detected in 19 patients (cM1: lungs, 10 cases; liver, 3 cases; bone, 4 cases; axillary LN, 1 case; pleurae 1 case). Among cM1 cases, 11 died of disease and 5 were alive with disease.

Discussion

The present study examined 304 SDC cases, which represents the largest cohort of SDC reported to date, and provides extensive insights into the clinical outcomes, treatment, and prognostic factors of SDC. The results obtained support an aggressive clinical course in spite of the lower rate of distant metastases (31%) than in Boon's

For Peer Review

15

retrospective study [4] and a median OS of 11.61 years. In the study by Boon, the number of positive LN was the only factor independently associated with poor OS and DMFS [4]. Previous studies reported that 5-year OS rates in patients with SDC ranged between 12 and 55%: the weighted average of five-year DFS and OS rates were 46 and 35%, respectively [9-17]. The majority of studies on the clinical outcome of SDC presented data from a single institution. However, Jayaprakash et al. [18] conducted an analysis of 228 patients using the Surveillance, Epidemiology, and End Results database. The findings obtained showed that the 10-year OS rate was 42% and median OS was 79 months, with the majority of deaths occurring within the first five years of the diagnosis of SDC [18]. Even in patients with early T stage SDC, the overall prognosis was poor (five-year DFS and OS rates of 49%) [16]. Otsuka et al. [5] reported 3-year OS and DFS rates of 70.5 and 38.2%, respectively, in 141 SDC cases from multiple institutions, showed that an advanced N stage independently affected both OS and DFS, and identified the most common treatment failure as distant metastasis. In the present study, the most common treatment failure in SDC patients was also distant metastasis. Although another analysis of a larger cohort (n=56) subsequently showed similar outcomes, with 3- and 5-year OS rates of 42.7 and 26.9%, respectively, recent studies with similar cohort sizes reported a better 5-year OS rate of 55.1%, suggesting

For Peer Review

the benefits of the intensification of both surgery and adjuvant RT for treatment outcomes [12,19,20]. However, marked differences were observed between OS and DFS; the 5-year DFS was 29% in one study [19], whereas Otsuka et al. [5] indicated 3-year OS and DFS rates of 70.5% and 38.2%, respectively. This discrepancy reflects the markedly high ratio of treatment failure for SDC. In the present study, 3-, 5- and 10-year CIR were 46.3, 49.0, and 57.4%, respectively (3-, 5-, and 10-year DFS rates were 48.5, 41.7, and 32.6%, respectively; data not shown). In our cohort, Three-year DFS was slightly better in the present study than previously reported [5,12,18,19], which may be attributed to advances in post-operative therapies.

In the present study, a higher pathological stage, which was associated with advanced T and N factors, and large numbers of cancer-positive LN were identified as independent prognostic factors. Boon et al. [4] and Otsuka et al. [5] indicated that advanced N factors and/or the number of positive LN correlated with OS and DFS or DMFS. In the present study, an advanced N factor (N0 vs N2/N3) and ≥11 cancer-positive LN correlated with poor 5-year OS, 5-year CIR, and 5-year CIDMR. These were consistent with previous studies [4,5]. SDC had higher incidences of LN and distant metastases than those reported by Osborn (46.5%) and Jayaprakash et al. (49%), respectively [18,21]. In the present study, outcomes were worse in cases with minor SG

For Peer Review

than in those with the parotid gland and submandibular gland as the primary tumor site. Since standard therapeutic strategies have not yet been established for SDC cases in which minor SG is the primary tumor site, and, thus, adequate therapies were not performed for these cases, their outcomes were worse. Furthermore, a negative surgical margin may not have been achieved in these cases, resulting in incomplete resection. Therefore, clinicians need to consider these factors in cases of SDC arising from minor SG.

In the statistical analyses, we mainly used competing-risk analysis, in which death was employed as a competing risk, to analyze the cumulative incidence of relapse, local relapse, LN metastasis, and distant metastasis in order to produce more precise statistical results. Kaplan-Meier curve analysis frequently leads to the cumulative risk that patients are exposed to being overestimated, and when a competing risk is present the cumulative risk of patients with certain diseases is not as high as the cumulative risk indicated by the Kaplan-Meier method [9,22,23].

Otsuka et al. [5] (n=141) and Jayaprakash et al. [18] (n=228) identified age and the N factor as independent prognostic factors for OS and DFS/disease-specific survival, in addition to the tumor size and grade in a multivariate analysis. However, a correlation was not observed between age and outcomes in the 304 SDC cases examined in the

For Peer Review

present study. However, LN metastasis (N[+]) was associated with worse OS, CIR, CICLNM, and CIDMR than N0 cases, and was one of the independent factors predicting a poor outcome.

In our cohort, the most common form of treatment failure was late distant metastases (n=93 in our series), which is consistent with the findings from smaller cohorts [11,20,24] and a larger cohort [5]. Previous studies identified the lungs and bone as the most common sites of distant metastasis in SDC [5,12,21,25], which is in accordance with the present results. A high ratio of distant metastases is presumed to be the leading cause of high CIR and CIDMR or low DFS and DMFS. Although extended resection with wider margins combined with intensified adjuvant RT appear to have contributed to better treatment outcomes in SDC patients by improving locoregional control, these strategies alone cannot prevent the development of delayed distant metastasis. Therefore, effective systemic therapy after curative surgery is imperative for improving CIR and CIDMR in SDC patients. Immunohistochemical studies revealed the expression of AR in 69-100% of SDC cases [25-27], whereas that of HER2 was only observed in 26-77%, both of which were confirmed in other reports, suggesting a potential role for agents targeting these receptors in molecular-targeted therapy for SDC [5,28,29]. Despite the focal or heterogenous expression of AR, androgen deprivation

For Peer Review

therapy (ADT) was found to be clinically beneficial for patients with AR-positive SDC, with 18% achieving a partial response and 50% stable disease in addition to longer DSF [30–32]. However, some cases acquire resistance to ADT due to the aberrant expression of SRD5A1 and loss of FOXA1 expression [33,34]. The administration of trastuzumab and docetaxel to patients with HER2-positive SDC achieved a good overall response (70.2%: 95%CI, 56.6-81.6%), including partial and complete responses, and was clinically beneficial (84.2%; 95%CI, 72.1-92.5%), with increases in OS and progression-free survival [35]. Since the status of patients with early or late distant metastasis is systemic, novel chemotherapy regimens are needed, such as ADT for AR-positive SDC and/or trastuzumab therapy for HER2-positive SDC [36]. Similar to our cohort, only a few patients have been treated with ADT or trastuzumab and, thus, the therapeutic effects of these agents remain unclear. AR, HER2, and EGFR profiles in SDC patients in our series are currently being investigated.

In the present study, the outcomes of SDC ex-PA-WI and *de novo* SDC were both poor, whereas that of SDC ex-PA-IC/MinI was better. Hashimoto's classification was used in the present study to stage CXPA [7] because the TNM classification focused on the extent of invasion of carcinoma and not the tumor size; since CXPA-IC cases may exhibit large tumors, and CXPA-WI cases small tumors. Since the extent of invasion of

For Peer Review

MinI CXPA markedly varies between 1.5 and 8 mm in the 4th WHO classification, we established MinI ≤2 mm from the fibrous capsule of a co-existing PA for a more practical and easily measurable value. Few studies have investigated differences between CXPA(-) and CXPA(+) cases [4,10]. Griffith et al. showed that OS was significantly worse in extracapsular invasive-type SDC ex-PA than in IC-type SDC ex-PA [37]. IC-type SDC ex-PA is an indolent tumor, whereas invasive-type SDC ex-PA is an aggressive tumor, similar to *de novo* SDC; therefore, WI-type SDC ex-PA need to be added to the analytical cohort. In our series, nine out of the 47 cases of IC-type SDC ex-PA died mainly due to other diseases except for one case. Therefore, IC-type SDC ex-PA has a better outcome than invasive SDC.

In conclusion, SDC frequently occurs in major SG, mostly in the parotid gland; however, outcomes are worse in minor SG cases than in major SG cases. A high N factor, particularly large numbers (11≥) of cancer-positive LN, or high pathological stage were identified as factors contributing to a worse prognosis, and the main reason for treatment failure was delayed distant metastases.

References

For Peer Review

1. Nagao T, Licitra L, Loening T, Vielh P, Williams MD. “Salivary duct carcinoma” (eds.) El-Nagger A, Chan JKC, Grandis JR, Takata T. Slootweg PJ. WHO classification of head and neck tumours. Lyon, IARCPress, 2017:173-174.
2. Williams MD, Ihrler S, Seethala R. “Carcinoma ex pleomorphic adenoma” (eds.) EL-Nagger A, Chan JKC, Grandis JR, Takata T. Slootweg PJ. WHO classification of head and neck tumours. Lyon, IARCPress, 2017: 176-177.
3. Kapadia SB, Barns L. Expression of androgen receptor, gross cystic disease fluid protein, and CD44 in salivary duct carcinoma. Mod Pathol 1998;11:1033-1038.
4. Boon E, Bel M, van Boxtel, et al. A clinicopathological study and prognostic factor analysis of 177 salivary duct carcinoma patients from the The Netherlands. Int J Cancer 2018;143: 758-766.
5. Otsuka K, Imanishi Y, Tada Y, et al. Clinical outcomes and prognostic factors for salivary duct carcinoma: A multi-institutional analysis of 141 patients. Ann Surg Oncol 2016; 23: 2038-2045.
6. Brierley JD, Gospodarowicz MK, Wittekind C, O’Sullivan B, Mason M, Asamura H, Lee A, Van Eycken E, Denny L, Amin MB, Gupta S. (eds.) Union for International Cancer Control: TNM Classification of Malignant Tumours: The 8th ed., John Wiley & Sons. Ltd. Sussex, UK. 2017: 47-50.

For Peer Review

22

7. Hashimoto K, Yamamoto H, Shiratsuchi H, et al. S100P expression in ductal type of carcinoma ex pleomorphic adenoma. Am J Surg Pathol 2011; 35: 346-365.

8. Kim HT. Cumulative incidence in competing risks data and competing risks regression analysis. Clin Cancer Res 2007; 13(2 Pt 1): 559-565.

9. Lacoppidan T, Vogelius IR, Pohl M, Strange M, Persson GF, Nygard L. An investigative expansion of a competing risk model for first failure site in locally advanced non-small cell lung cancer. Acta Oncol 2019; 58: 1386-1392.

10. Schmitt NC, Kang H, Sharma A. Salivary duct carcinoma: An aggressive salivary gland malignancy with opportunities for targeted therapy. Oral Oncol 2017; 74: 40-48.

11. Breinholt H, Elhakim MT, Godballe C, et al. Salivary duct carcinoma: A Danish national study. J Oral Pathol Med 2016; 45: 664-667.

12. Kim JY, Lee S, Cho KJ, et al. Treatment results of post-operative radiotherapy in patients with salivary duct carcinoma of the major salivary glands. Br J Radiol 2012; 85: e947-e952.

13. Guzzo M, Di Palma S, Grandi C, Molinari R. Salivary duct carcinoma: Clinical characteristics and treatment strategies. Head Neck 1997; 19: 126-133.

For Peer Review

14. Shinoto M, Shioyama Y, Nakamura K, et al. Postoperative radiotherapy in patients with salivary duct carcinoma: clinical outcomes and prognostic factors. J Radiat Res 2013; 54: 925-930.

15. Salovaara E, Hakala O, Back L, et al. Management and outcome of salivary duct carcinoma in major salivary glands. Eur Arch Otorhinolaryngol 2013; 270: 281-285.

16. Schimitt NC, Sharma A, Gilbert MR, Kim S. Early T stage salivary duct carcinoma: outcomes and implications for patient counseling. Otolaryngol Head Neck Surg 2015; 153: 795-798.

17. Villepelt A, Lefevre M, Verillaud B, et al. Salivary duct carcinoma: Prospective multicenter study of 61 cases of the Reseau d'Expertise Francais des Cancers ORL Rares. Head Neck 2018; 41: 584-591.

18. Jayaprakash V, Merzianu M, Warren GW, et al. Survival rates and prognostic factors for infiltrating salivary duct carcinoma: analysis of 228 cases from the Surveillance, Epidemiology, and End Results database. Head Neck 2014; 36: 694-701.

For Peer Review

19. Roh JL, Lee JI, Choi SH, et al. Prognostic factors and oncologic outcomes of 56 salivary duct carcinoma patients in a single institution: high rate of systemic failure warrants targeted therapy. Oral Oncol 2014; 50: e64-e66.

20. Johnston ML, Huang SH, Waldron JN, et al. Salivary duct carcinoma: Treatment, outcomes, and patterns of failure. Head Neck 2016; 38 (Suppl 1): E820-E826.

21. Osborn V, Givi B, Lee A, et al. Characterization, treatment and outcomes of salivary duct carcinoma using the National Cancer Database. Oral Oncol 2017; 7: 41-46.

22. Yang J, Pan Z, He Y, et al. Competing-risk model for predicting the prognosis of penile cancer based on the SEER database. Cancer Med 2019; 8: 7881-7889.

23. Yu X, Gao S, Xue Q, et al. Development of a predictive nomogram for cause-specific mortality in surgically resected early-stage oesophageal cancer: a Surveillance, Epidemiology, and End Results (SEER) analysis. J Thorac Dis 2020; 12: 2583-2594.

24. Gilbert MR, Sharma A, Schmitt NC, et al. A 20-year review of 75 cases of salivary duct carcinoma. JAMA Otolaryngol Head Neck Surg 2016; 142: 489-495.

For Peer Review

25

25. Jaehne M, Roeser K, Jaeket T, Schepers JD, Albert N, Loning T. Clinical and immunohistochemical typing of salivary duct carcinoma: a report of 50 cases. Cancer 2005; 103: 2526-2533.

26. Locati, LD, Perrone F, Losa M, et al. Treatment relevant target immunophenotyping of 139 salivary gland carcinomas (SGCs). Oral Oncol 2009; 45: 986-900.

27. Kapadia SB, Barnes L. Expression of androgen receptor, gross cystic disease fluid protein, and CD44 in salivary duct carcinoma. Mod Pathol 1998; 11: 1033-1038.

28. Fan CY, Wang J, Barnes EL. Expression of androgen receptor and prostatic specific markers in salivary duct carcinoma: an immunohistochemical analysis of 13 cases and review of the literature. Am J Surg Pathol 2000; 24: 579-586

29. Xu B, Dogan S, Haroon Al Rasheed MR, Ghossein R, Katabi N. Androgen receptor immunohistochemistry in salivary duct carcinoma: A retrospective study 188 cases focusing on tumor heterogeneity and temporal concordance. Hum Pathol 2019; 93: 30-36.

For Peer Review

30. Williams MD, Roberts DB, Kies MS, Mao L, Weber RS, El-Nagger AK. Genetic and expression analysis of HER-2 and EGFR genes in salivary duct carcinoma: empirical and therapeutic significance. Clin Cancer Res 2010; 16: 2266-2274.

31. van Boxtel W, Locati LD, van Engen-van Grunsven ACH et al. Adjuvant androgen deprivation therapy for poor risk, androgen receptor-positive salivary duct carcinoma. Eur J Cancer 2019; 110: 62-70.

32. Boon E, van Boxtel W, Buter J, et al. Androgen deprivation therapy for androgen receptor-positive advanced salivary duct carcinoma: A nationwide case series of 35 patients in The Netherlands. Head Neck 2018; 40: 605-613.

33. Fushime C, Tada Y, Takahashi H, et al. A prospective phase II study of combined androgen blockage in patients with androgen receptor-positive metastatic or locally advanced unresectable salivary gland carcinoma, Ann Oncol 2018; 29: 979-983.

34. Locati LD, Perrone F, Cartelazz B et a. Activity of abiraterone in rechallenging two AR-expressing salivary gland adenocarcinoma, resistant to androgen deprivation therapy. Cancer Biol Ther 2014; 15: 678-682.

For Peer Review

27

35. Wasano K, Sakurai K, Kawasaki T, et al. Acquisition of resistance to androgen deprivation therapy in salivary duct carcinoma: A case report. Rare Tumor 2018; 10: 1-5.

36. Takahashi H, Tada Y, Saotome T, et al. Phase II trial of Trastuzumab and Docetaxel in patients with human epidermal growth factor receptor-2-positive salivary duct carcinoma. J Clin Oncol 2019; 37: 125-134.

37. Ujien MJM, Lassche G, van Engen-van-Grunsven ACH, et al. Systemic therapy in the management of recurrent or metastatic salivary duct carcinoma: A systemic review. Cancer Treat Rev 2020; 89: 102069 doi: 10.1016/j.ctrv2020,102069.

38. Griffith CC, Thompson LD, Assaad A, et al. Salivary duct carcinoma and the concept of early carcinoma ex pleomorphic adenoma. Histopathology 2014; 65: 854-860.

For Peer Review

Figure legends

Figure 1. Consort diagram of the inclusion of SDC cases. All data were collected from 18 institutions and consultation cases (K.K.) and 304 eligible cases of SDC were ultimately selected.

Figure 2 (A) Overall survival (OS), (B) disease-free survival curve (DFS), and (C) distant metastasis-free survival (DMFS) in 304 patients with SDC. The non-dotted line represents survival probability and dotted lines show the 95% confidence interval. Three- and five-year OS, DFS, and DMFS rates were 77.9 and 64.6%, 48.5 and 41.7%, and 53.5 and 45.8%, respectively.

Figure 3. Cumulative incidence of relapse (CIR) curves according to each prognostic factor identified in the univariate analysis and multivariate Fine-Grey proportional hazard regression model. CIR according to the site (A) ($p<0.001$), pStage (B) ($p<0.001$) and number of LN metastasis (C) ($p<0.001$).

For Peer Review

29

Figure 4. Cumulative incidence of distant metastasis relapse (CIDMR) curves according to each prognostic factor identified in the univariate analysis and multivariate Fine-Grey proportional hazard regression model. CIDMR according to the site (A) ($p=0.0476$), pStage (B) ($p<0.001$), and number of LN metastasis (C) ($p<0.001$).

Figure 5. Patterns of disease recurrence. (A) Local and regional recurrence and distant metastases in 110 patients with recurrence. The numbers in the circles represent the absolute number of patients with local and regional recurrence and the presence of distant metastases. Patients with primarily metastatic disease were not included in this figure. (B) Localization of distant metastases sorted by absolute numbers in 93 patients with distant metastases. Patients with primarily metastatic diseases were not included in this figure.

Supplemental figures

Supplemental Figure 1. (A) Typical histology of a *de novo* (CXPA[-]) case showing Roman bridge structures of large atypical cells with an eosinophilic cytoplasm, and

For Peer Review

comedonecrosis (hematoxylin & eosin stain). (B) Typical histology of a CXPA(+) case showing the co-existence of a pleomorphic adenoma (PA) circumscribed with a fibrous capsule (yellow dotted line). The intracapsular component (IC) showed the growth of atypical glandular cells within the PA component, whereas the invasive component (Inv) showed the extracapsular growth of SDC cells (hematoxylin & eosin stain).

Supplemental Figure 2. Cumulative incidence of relapse (CIR) (A), cumulative incidence of local relapse (CILR) (B), cumulative incidence of cervical lymph node relapse (CICLNR) (C), and cumulative incidence of distant metastasis relapse (CIDMR) (D). The non-dotted line represents each incidence and dotted lines show the 95% confidence interval.

For Peer Review

Figure 1

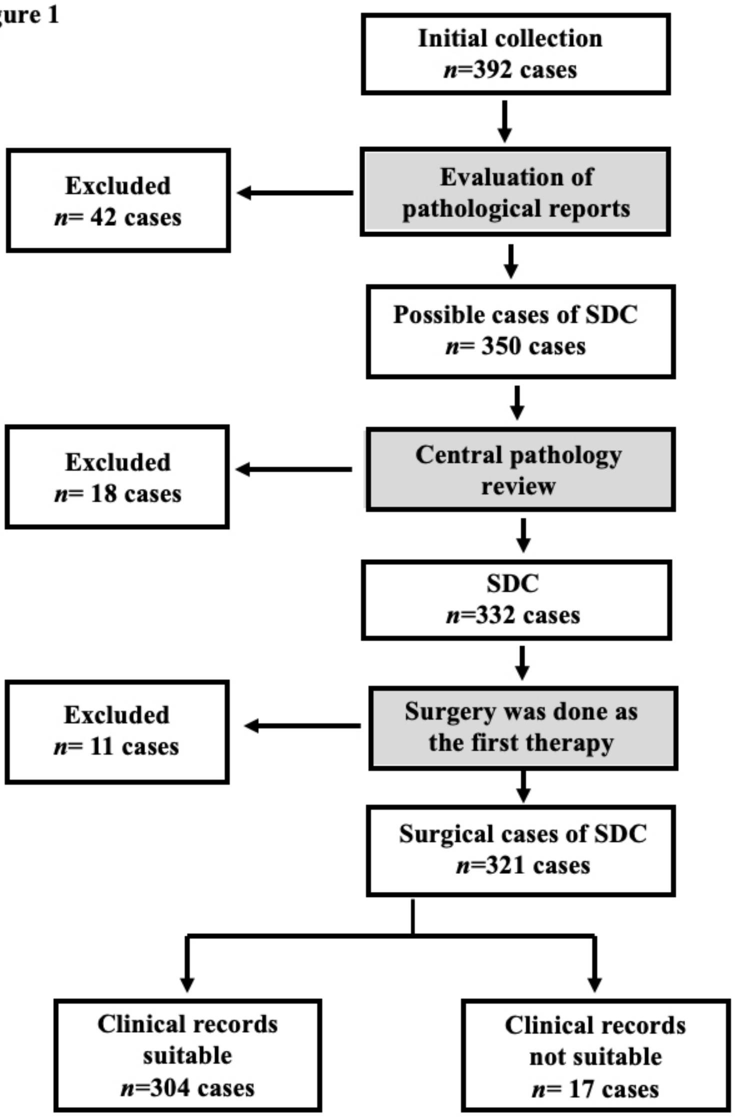

Figure 1. Consort diagram of inclusion of SDC patients. All data were collected from 18 institutions and consult cases (K.K.) and according to this diagram, eligible 304 cases of SDC were finally selected.

190x254mm (96 x 96 DPI)

Figure 2

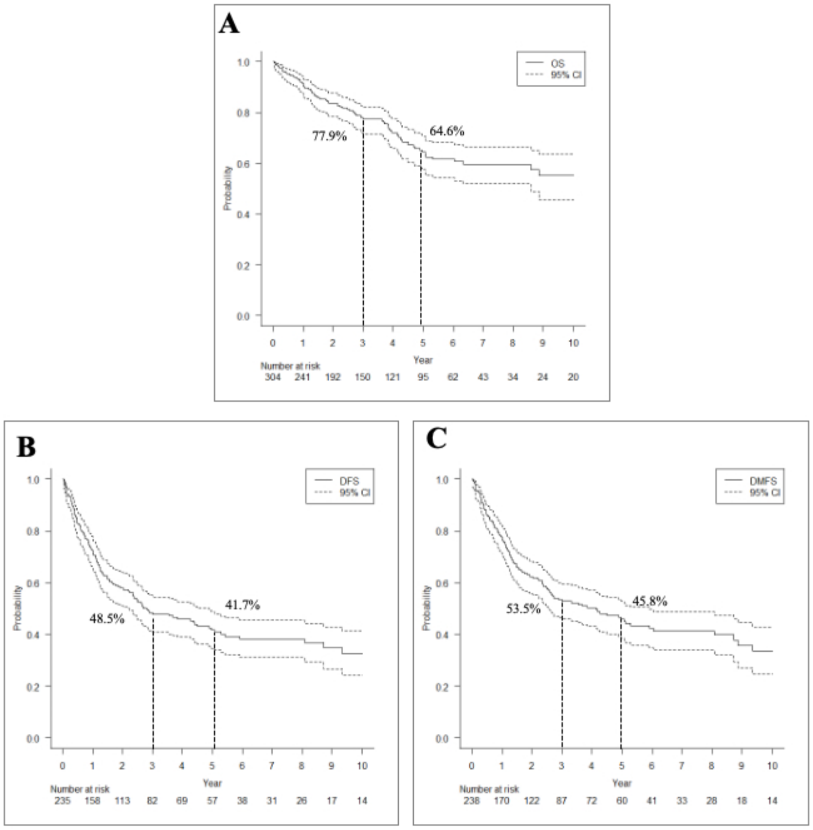

Figure 2 (A) Overall survival (OS), (B) disease free survival curve (DFS) and (C) distant metastasis free survival (DMFS) of all 304 patients with SDC. The non-dotted line represents the survival probability and the dotted lines represents the 95% confidence interval. The 3-year and 5-year OS, DFS and DMFS rates were 77.9% and 64.6%, 48.5% and 41.7 %, and 53.5% and 45.8%, respectively.

190x254mm (96 x 96 DPI)

Figure 3

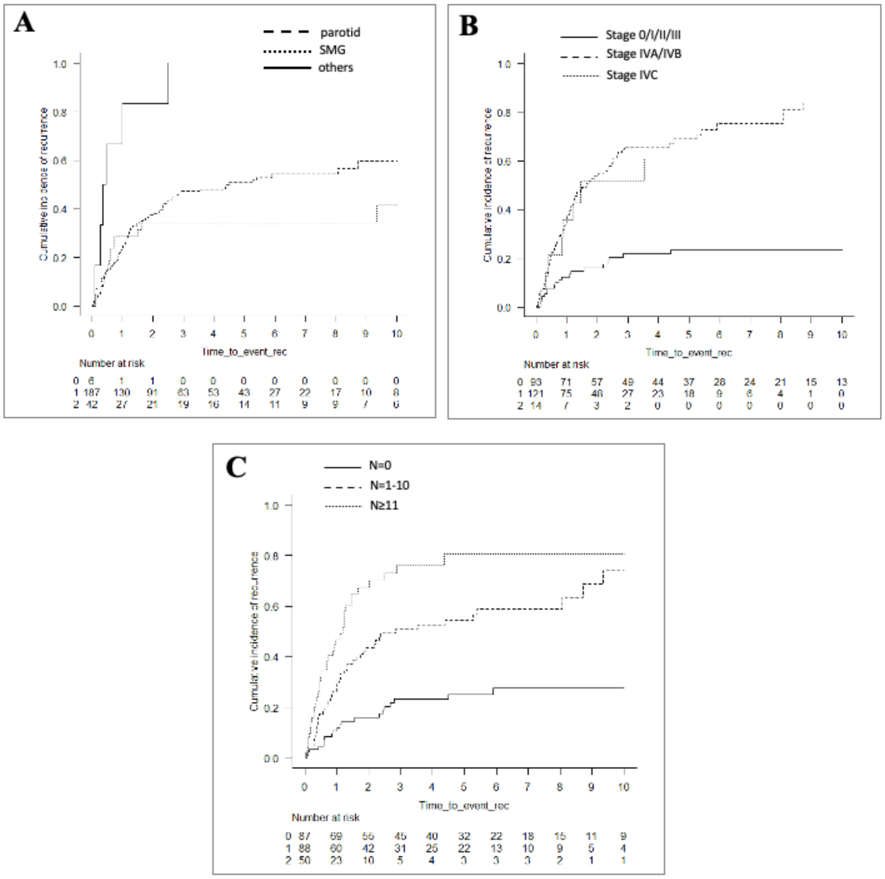

Figure 3. The cumulative incidence of relapse (CIR) curves according each of the prognostic factors that were found to be significant on both univariate analysis and multivariate Fine-Grey proportional hazard regression model are shown as follows: CIR according to the site (A) ($p<0.001$), Stage (B) ($p<0.001$) and numbers of LN metastasis (C) ($p<0.001$), respectively.

190x254mm (96 x 96 DPI)

Figure 4

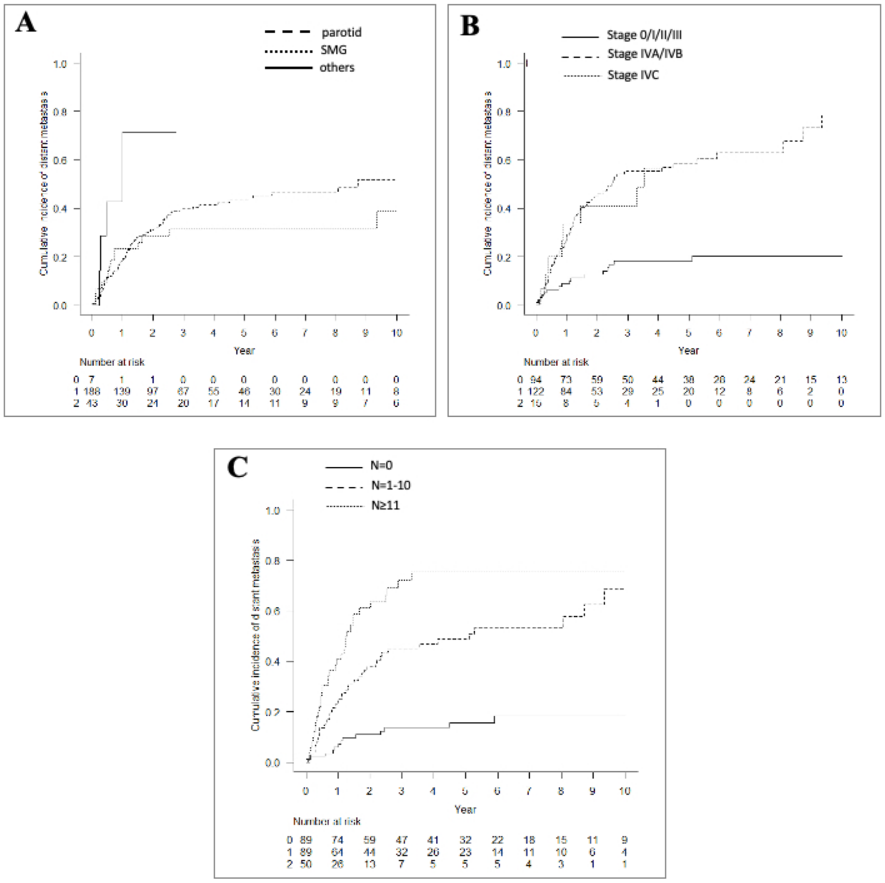

Figure 4. The cumulative incidence of distant metastasis relapse (CIDMR) curves according each of the prognostic factors that were found to be significant on both univariate analysis and multivariate Fine-Grey proportional hazard regression model are shown as follows: CIDMR according to the site (A) (p=0.0476), Stage (B) (p<0.001) and numbers of LN metastasis (C) (p<0.001), respectively.

190x254mm (96 x 96 DPI)

Figure 5

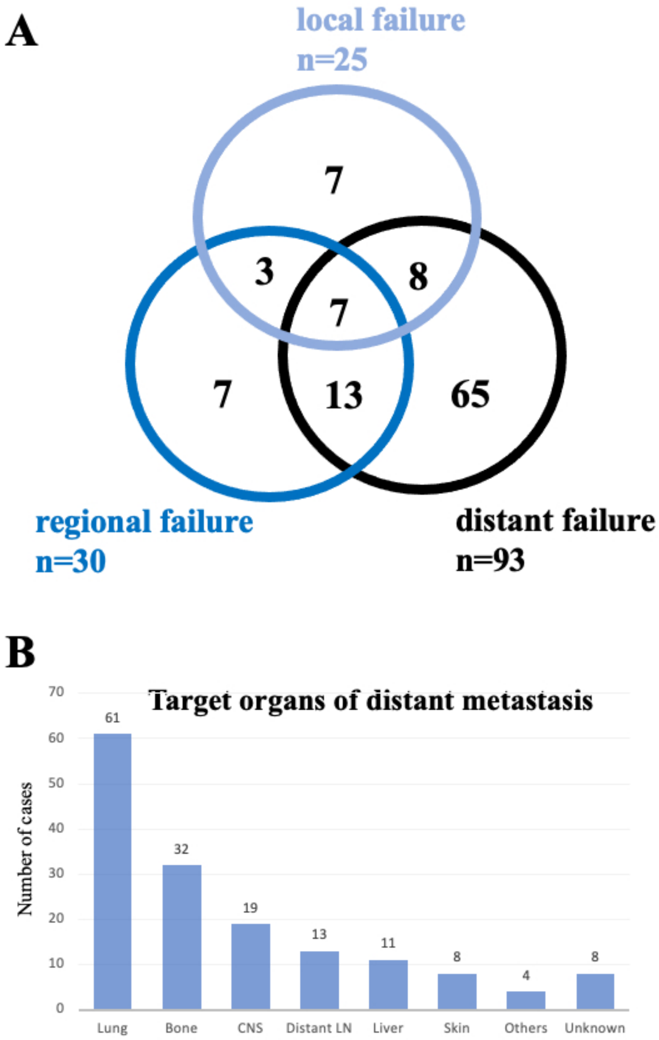

Figure 5. Patterns of disease recurrence. (A) Breakdown of local and regional recurrences and distant metastases in 110 patients with a recurrence. The numbers in the circles represent the absolute number of patients with local and regional recurrences and the presence of distant metastases. Patients with primarily metastatic disease are not included in this figure. (B) Localization of distant metastases sorted by absolute numbers of presence in 93 patients with distant metastases. Patients with primarily metastatic diseases are not included in this figure.

190x254mm (96 x 96 DPI)

Table 1. Characteristics of 304 patients with salivary duct carcinoma

	No. of patients	percentage (%)
**Age(y); median=68 (27-91)**		
**≤49**	34	11
**50-59**	53	17
**60-69**	99	33
**70-79**	81	27
**≥80**	37	12
**Gender**		
**male**	253	83
**female**	51	17
**Site**		
**parotid gland**	238	78
**SMG**	55	18
**others**	11	3.6
**CXPA**		
**CXPA(-)/de novo cancer**	121	40
**CXPA(+): IC**	47	15
**CXPA(+): MinI**	23	7.6
**CXPA(+): WI**	112	37
**unknown**	1	0.3
**StageT factor**		
**Stage 0 Tis**	28	0.72.6
**Stage I T1**	5861	1920
**Stage II T2**	3371	1123
**Stage III T3**	4579	1526
**Stage IVA T4**	11880	3926
**Stage IVB Tx**	192	60.7
**Stage IVCN factor**	19	6
**unknown N0**	10131	3.343
**No. of LN metastasis N1**	36	12
**0 N2**	126108	4136
**1-10 N3**	10215	345
**≥11 N(+)**	572	190.7
**unknown Nx**	1912	64

For Peer Review

**Therapy~~M factor~~**		
**S ~~M0~~**	107~~281~~	35~~92~~
**S+POT ~~M1~~**	197~~19~~	65~~6~~

SMG, submandibular gland; CXPA, carcinoma ex pleomorphic adenoma;

IC, intracapsular; MinI, minimally invasive; WI, widely invasive;

No., number; LN, lymph node; S, surgery; POT, post-operative therapy.

*N does not include the late cervical LN metastases.

**M does not include the late distant metastases.

For Peer Review

Table 1. Characteristics of 304 patients with salivary duct carcinoma

	No. of patients	percentage (%)
**Age(y); median=68 (27-91)**		
**≤49**	34	11
**50-59**	53	17
**60-69**	99	33
**70-79**	81	27
**≥80**	37	12
**Gender**		
**male**	253	83
**female**	51	17
**Site**		
**parotid gland**	238	78
**SMG**	55	18
**others**	11	3.6
**CXPA**		
**CXPA(-)/de novo cancer**	121	40
**CXPA(+): IC**	47	15
**CXPA(+): MinI**	23	7.6
**CXPA(+): WI**	112	37
**unknown**	1	0.3
**Stage**		
**Stage 0**	2	0.7
**Stage I**	58	19
**Stage II**	33	11
**Stage III**	45	15
**Stage IVA**	118	39
**Stage IVB**	19	6
**Stage IVC**	19	6
**unknown**	10	3.3
**No. of LN metastasis**		
**0**	126	41
**1-10**	102	34
**≥11**	57	19
**unknown**	19	6

For Peer Review

**Therapy**		
**S**	107	35
**S+POT**	197	65

SMG, submandibular gland; CXPA, carcinoma ex pleomorphic adenoma;

IC, intracapsular; MinI, minimally invasive; WI, widely invasive;

No., number; LN, lymph node; S, surgery; POT, post-operative therapy.

*N does not include the late cervical LN metastases.

**M does not include the late distant metastases.

For Peer Review

Table 2. Univariate analyses for overall survival, cumulative incidence of recurrence and cumulative incidence of distant metastasis

			OS		CIR		CIDMR	
		N	HR (95%) CI	*p*-value	HR (95%) CI	*p*-value	HR (95%) CI	*p*-value
**Age**	<65y/o	131	0.83 (0.55-1.26)	0.380	0.79 (0.54-1.14)	0.200	0.83 (0.56-1.25)	0.27
	≥65y/o.	173	Ref.		Ref.		Ref.	
**Gender**	female	51	Ref.		Ref.		Ref.	
	male	252	2.05 (1.03-4.09)	**0.041**	1.68 (0.94-3.01)	0.081	1.88 (0.98-3.6)	0.058
**Site**	parotid	238	2.33 (0.32-16.76)	0.402	0.21 (0.1-0.44)	**<0.001**	0.33 (0.11-1)	0.050
	SMG	55	1.68 (0.22-	0.617	0.14 (0.05-	**<0.001**	0.24 (0.07-	**0.023**

			12.79)		0.44)		0.82)	
	others	11	Ref.		Ref.		Ref.	
**CXPA**	(-) de novo	121	Ref.		Ref.		Ref.	
	(+) IC/MinI	70	0.6 (0.32-1.1)	0.098	0.63 (0.32-1.25)	0.190	0.64(0.32-1.31)	0.220
	(+) WI	112	1.12 (0.72-1.75)	0.615	0.91 (0.62-1.34)	0.630	0.77 (0.5-1.17)	0.220
**T**	Tis/pT1	69	Ref.		Ref.		Ref.	
	T2/3	150	2.15(1.11-4.15)	**0.023**	2.41 (1.11-5.24)	**0.026**	3.44 (1.27-9.27)	**0.015**
	T4	80	3.39 (1.69-6.82)	**<0.001**	4.84(2.24-10.45)	**<0.001**	6.29 (2.23-17.06)	**<0.001**
**N**	N0	131	reference		reference		reference	
	N1	36	0.98	0.958	2.21	**0.018**	3.05	**0.003**

			(0.43-2.24)		(1.15-4.25)		(1.48-6.26)	
	N2/N3/N(+)	123	2.9 (1.82-4.63)	**<0.001**	4.07(2.52-6.59)	**<0.001**	5.23 (2.98-9.18)	**<0.001**
**M**	M0	281	Ref.		Ref.		Ref.	
	M1	19	2.578(1.51-5.12)	**<0.001**	1.32 (0.64-2.74)	0.460	1.43 (0.69-2.95	0.330
**Stage**	Stage 0/I/II/III	138	Ref.		Ref.		Ref.	
	Stage IVA/B	137	3.38 (2.05-5.6)	**<0.001**	4.86 (2.9-8.14)	**<0.001**	4.25 (2.47-7.32)	**<0.001**
	Stage IVC	19	5.61 (2.77-11.35)	**<0.001**	3.56(1.5-8.14)	**0.004**	3.6 (1.52-8.53)	**0.004**
**No. of**	0	126	Ref.		Ref.		Ref.	**0.001**

**LN metastasis**								
	1-10	102	1.87 (1.1-3.15)	**0.020**	2.94 (1.78-4.88)	**<0.001**	4.02 (2.23-7.27)	**<0.001**
	≥11	57	4.14 (2.41-7.11)	**<0.001**	5.39 (3.09-9.39)	**<0.001**	7.32 (3.88-13.81)	**<0.001**
**Therapy**	S	107	Ref.		Ref.		Ref.	
	S+POT	197	1.1(0.7-1.74)	0.669	1.63 (0.99-2.69)	0.055	2.27 (1.27-4.05)	0.006

Bold shows p<0.05.

HR, hazard ratio; 95%CI, 95% confidence interval; OS, overall survival; CIR, cumulative incidence of recurrence; CIDMR, cumulative incidence of distant metastasis relapse; No, number; Ref., reference; CXPA, carcinoma ex pleomorphic

adenoma; IC, intracapsular type; MinI, minimally invasive type; WI, widely invasive type; SMG, submandibular gland; LN, lymph node; S, surgery; POT, post-operative therapy.

For Peer Review

Table 3. Multivariate analysis for overall survival, cumulative incidence of recurrence and cumulative incidence of distant metastasis

	**OS**		**CIR**		**CIDMR**	
	**HR (95% CI)**	***p*-value**	**HR (95% CI)**	***p*-value**	**HR (95% CI)**	***p*-value**
**Gender**						
**male**	1.54 (0.76-3.09)	0.230	ND	ND	ND	ND
**female**	Ref.		ND	ND	ND	ND
**Site**						
**parotid gland**	ND	ND	0.28 (0.16-0.52)	**<0.001**	0.28 (0.14-0.59)	**<0.001**
**SMG**	ND	ND	0.27 (0.12-0.63)	**0.003**	0.3 (0.12-0.77)	**0.012**
**others**	ND	ND	Ref.		Ref.	
**Stage**						
**Stage 0/I/II/III**	Ref.		Ref.		Ref.	

**Stage IVA/B**	2.65 (1.44-4.88)	**0.002**	3.35 (1.83-6.14)	**<0.001**	2,42 (1.3-4.49)	**0.005**
**Stage IVC**	3.81 (1.73-8.41)	**<0.001**	2.25 (0.87-5.85)	0.096	1.92 (0.76-4.91)	0.170
**No. of LN metastasis**						
**0**	Ref.		Ref.		Ref.	
**1-10**	1.09 (0.59-2.02)	0.777	1.75 (1.03-2.99)	**0.040**	2,73 (1.45-5.14)	**0.002**
**≥11**	2,07 (1.08-3.94)	**0.028**	2.86 (1.57-5.2)	**<0.001**	4.63 (2.33-9.22)	**<0.001**

Bold shows p<0.05.

HR, hazard ratio; 95% CI, 95% confidence interval; OS, overall survival; CIR, cumulative incidence of recurrence; CIDMR, cumulative incidence of distant metastasis relapse; Ref., reference; SMG, submandibular gland; LN, lymph node; No, number; ND, not done.

*p-value of Wald's test relating to "recurrence coefficient=0"

For Peer Review

Supplemental figure 1

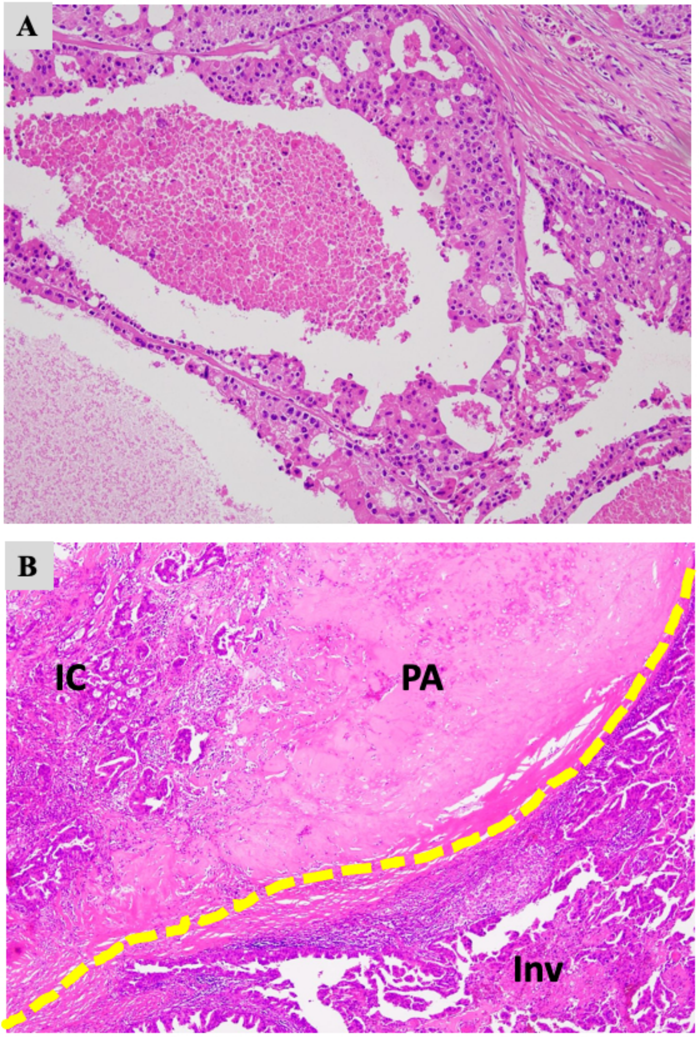

190x254mm (96 x 96 DPI)

Supplemental figure 2

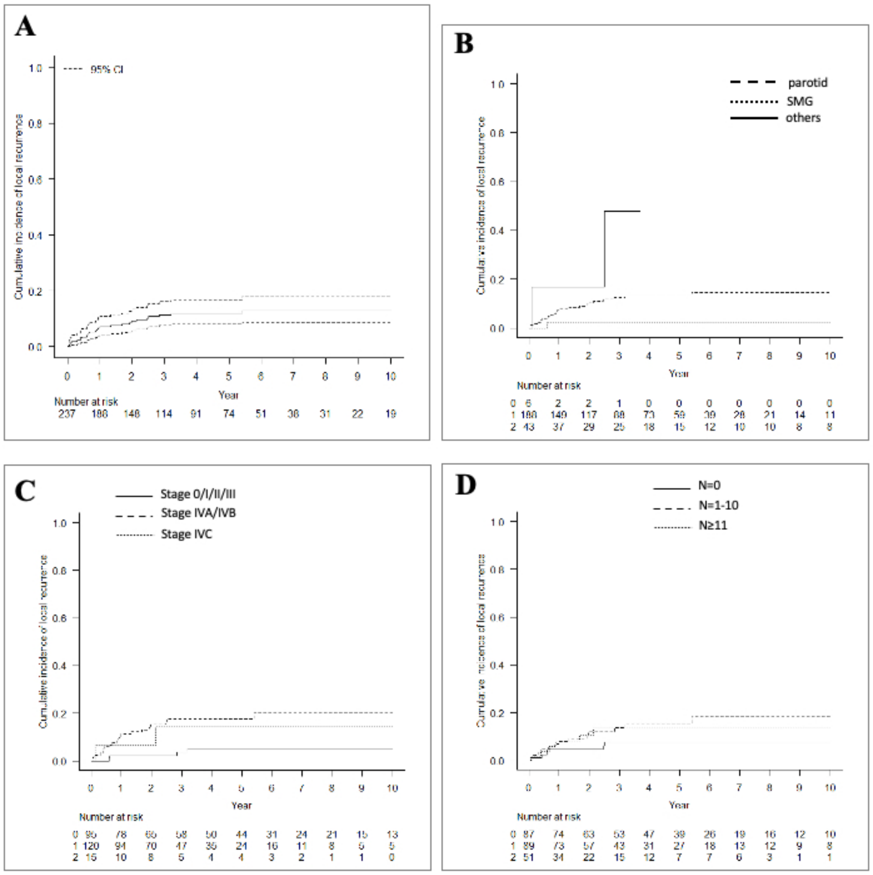

190x254mm (96 x 96 DPI)

Supplemental figure 3

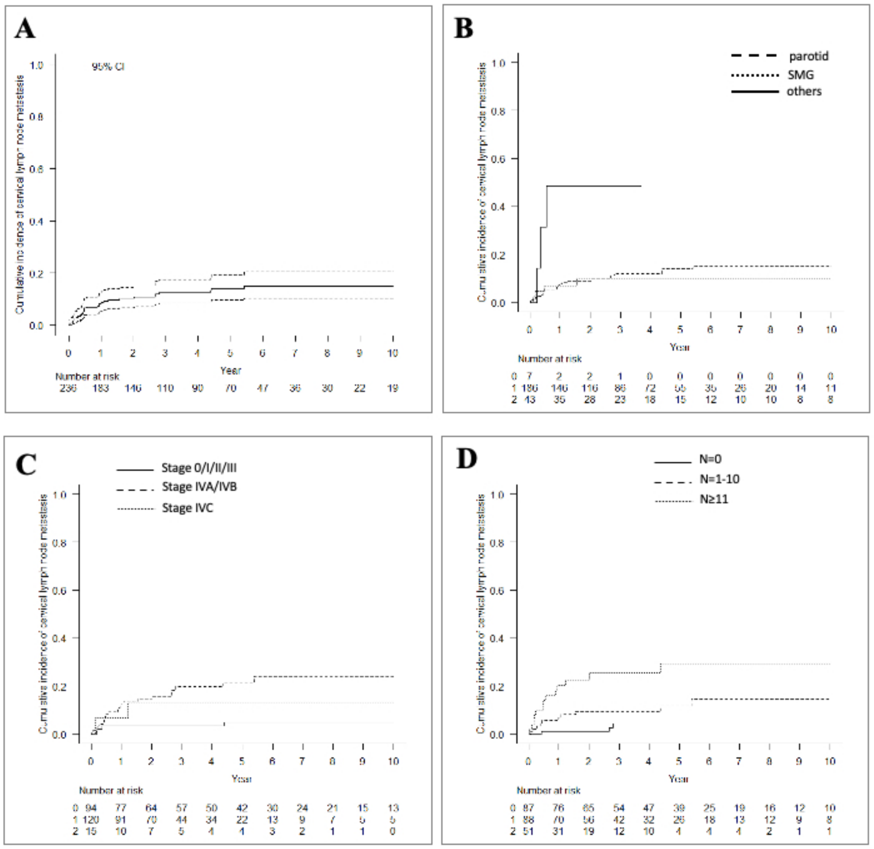

190x254mm (96 x 96 DPI)

Supplemental table 1. The summary of post-operative therapy (POT).

	No. of patients (n=197)	percentage
**S→RT**	102	52%
**S→Ch**$^{*/**}$	13	6.6%
**S→CRT****	70	36%
**S→S**$^{\#}$	5	2.5%
**S→unknown**	7	3.6%

S, surgery; RT, radiotherapy; Ch, chemotherapy; CRT, chemoradiotherapy.

*Including TS-1 administration (2 cases) and S-1 administration (1 case)

**Including Trastuzumab administration (6 cases), Nivolumab administration (3 case), and androgen deprivation therapy (5 cases).

#Including additional resection for local recurrence (2 cases), neck dissection (1 case), resection for distant metastasis (3 cases) and addition resection for recurrence (unknown location) (2 case).

For Peer Review

Supplemental table 2. Univariate and multivariate analyses for cumulative incidence of local relapse (CILR)and cumulative incidence of cervical lymph node metastasis (CICLNM)

		**Univariate analysis**				**Multivariate analysis**			
		**CILR**		**CICLNM**		**CILR**		**CICLNM**	
	**No**	**HR (95%) CI**	***p*-value**	**HR (95%) CI**	***p*-value**	**HR (95%) CI**	***p*-value**	**HR (95%) CI**	***p*-value**
**Age**									
**<65y**	131	0.87 (0.41-1.87)	0.720	0.68 (0.34-1.37)	0.280	ND		ND	
**≥65y**	173	Ref.		Ref.		ND		ND	
**Gender**									

**male**	253	1.08 (0.37-3.13)	0.890	1.04 (0.4-2.67)	0.940	ND		ND	
**female**	51	Ref.		Ref.		ND		ND	
**Site**									
**parotid**	238	0.25 (0.06-0.98)	0.047	0.16 (0.05-0.55)	**0.003**	0.34 (0.08-1.34)	**0.120**	0.26 (0.09-0.81)	**0.020**
**SMG**	55	0.04 (0-0.47)	**0.010**	0.11 (0.02-0.53)	**0.006**	0.08 (0.01-0.78)	0.030	0.2 (0.04-1.02)	0.053
**others**	11	Ref.	**0.027**	Ref.	**0.009**	Ref.	0.084	Ref.	0.057
**CXPA**									
**(-)/de**	12	Ref.	0.64	Ref.	0.335	ND		ND	

**novo cancer**	1		6				
**(+)IC/ MinI**	70	1.29 (0.59 - 2.83)		1.99 (0.72- 5.51)	**0.003**	ND	ND
**(+)WI**	112	1.26 (0.76 - 2.11)		1.63 (0.74- 3.6)	0.230	ND	ND
**T**							
**Tis /1**	69	Ref.	0.095	Ref.	0.262	ND	ND
**T2/3**	150	3.27 (0.45 - 24.04)		1.14 (0.32- 4.02)	0.840	ND	ND

**T4**	80	6.14 (0.84 - 45.08)		2.02 (0.56-7.31)	0.280	ND	ND
**N**							
**N0**	131	Ref.	0.099	Ref.	**0.003**	ND	ND
**N1**	36	1.35 (0.34 -5.3)	0.670	2.91 (0.59-14.33)	0.190	ND	ND
**N2/3**	123	2.62 (1.03 - 6.65)	0.043	7.2 (2.15-24.18)	**0.001**	ND	ND
**M**							
**M0**	281	1.16 (0.27	0.840	0.95 (0.22-	0.940	ND	ND

		-4,93)		4.06)					
**M1**	19	Ref.		Ref.		ND		ND	
**Stage**									
**0/I/II / III**	138	Ref.		Ref.	**0.009**	Ref.	0.052	Ref.	0.268
**IVA /B**	137	4.33 (1.5-12.46)	0.007	5.28 (1.82-15,35)	**0.002**	3.56 (1.28-9.93)	**0.015**	3.42 (0.71-16.55)	0.130
**IVC**	19	3.11 (0.57-16.95)	0.190	3.11 (0.54-17.31)	0.200	2.53 (0.48-13.5)	0.280	2.06 (0,27-15.85)	0.490
**No. of LN meta.**									

**0**	126	Ref.	0.279	Ref.	**0.001**	ND.	Ref.	0.095
**1-10**	102	2.19 (0.83-5.78)	0.110	3.9 (1.1-13.81)	**0.035**		2.24 (0.49-10.21)	0.300
**≥11**	57	1.84 (0.59-5.7)	0.290	9.21 (2.67-31.72)	**<0.001**		4.65 (0.92-23.4)	0.062
**Therapy**								
**S**	107	Ref.		Ref.		ND	ND	
**S+ POT**	197	0.56 (0.26-1.24)	0.273	0.93 (0.43-2.03)	0.850	ND	ND	

Bold shows $p<0.05$.

HR, hazard ratio; 95%CI, 95% confidence interval; CILR, cumulative incidence of

local relapse; CICLNM, cumulative incidence of cervical lymph node metastasis; Ref., reference; No, number; CXPA, carcinoma ex pleomorphic adenoma; IC, intracapsular type; MinI, minimally invasive type; WI, widely invasive type; SMG, submandibular gland; LN, lymph node; S, surgery; POT, post-operative therapy; ND, not done.

For Peer Review
